# Ascaris Lumbricoides Infestation as an Unexpected Cause of Acute Pancreatitis

**DOI:** 10.7759/cureus.12103

**Published:** 2020-12-15

**Authors:** Taimoor Hussain, Khalida Walizada, Tuba Khan, Rajeswari Khan, Zahra Mushtaq

**Affiliations:** 1 Neurology, Bolan Medical College, Quetta, PAK; 2 Neurological Surgery, Ali Abad Teaching Hospital, Kabul, AFG; 3 Medicine and Surgery, Ziauddin Medical College, Karachi, PAK; 4 Medicine and Surgery, College of Medicine & Sagore Dutta Hospital, Kolkata, IND; 5 Internal Medicine, Sandeman Provincial Hospital, Quetta, PAK

**Keywords:** ascariasis, acute pancreatitis, pakistan, balochistan, quetta, ascariasis-induced pancreatitis

## Abstract

Ascariasis is the most common helminthic infection. It is most common in children of tropical and developing countries where the transmission is by contamination of soil by human feces or use of untreated feces as fertilizer. Transmission in most endemic areas is via person to person contact. We hereby present a case report of acute pancreatitis due to ascariasis. Twenty-five-year-old male patient presented to us with chief complaint of acute epigastric pain radiating to back and associated with vomiting. Initial lab investigations revealed increased serum amylase and lipase. Probable diagnosis of acute pancreatitis was made. CT scan was done and report revealed bulky pancreas, and significant peri-pancreatic fat stranding. He was managed symptomatically with intravenous fluids, analgesics, anti-emetics and enteral nutrition. However, the cause remained undetermined as we ruled out the possible etiologies of acute pancreatitis until one day the patient vomited a 15-cm round worm. Thereafter, his condition improved dramatically. This is one of the first few case reports of ascariasis-induced pancreatitis from Pakistan and the first one from Baluchistan province of Pakistan. Thus it highlights ascariasis as possible etiology of acute pancreatitis in regions where ascariasis is geographically endemic.

## Introduction

Ascaris lumbricoides is one of the most common helminthic infections worldwide. With an overall prevalence of 25%, an estimated 1.4 billion people are infected and 1.2 to 2 million cases of clinical disease occur per year with around 20,000 deaths [1]. This is particularly prevalent in areas with poor sanitation, hot and humid climate areas of tropical and subtropical. Children between ages of 2 to 10 years are most commonly affected and prevalence decreases after the age of 15 years. The life cycle starts after ingestion of the eggs from contaminated food, soil, vegetables and water. After hatching larvae emerge in the duodenum and then migrate to cecum and through portal and systemic circulation reach the liver. They then migrate to the alveoli and ascend the bronchial tree and throat. Here they are swallowed and mature into adult worms [1]. As stated by Klimovskij et al. “the roundworms are actively motile, have wandering nature, and can migrate from their natural habitat in the duodenum and proximal jejunum into the ampulla of Vater and enter the bile duct or pancreatic duct causing cholangitis or pancreatitis, respectively” [2]. Clinical symptoms vary based on region of the body affected. Symptoms include “cough, ascariasis pneumonia, status asthmaticus needing ICU admission, nausea, vomiting, hepatobiliary and intestinal obstruction, stunting of growth, cognitive dysfunction and malnutrition in children with high ascaride load” [1].

In addition to gall stone and alcohol abuse which are the two major causes of acute pancreatitis, other etiologies include trauma, post endoscopic-retrograde cholangiopancreatography (ERCP), medications, hypercalcemia, hypertriglyceridemia, and infectious etiologies to name a few [3]. We present here a case of 25-year-old male resident of Quetta, Pakistan. He came to the emergency department with chief complaints of epigastric pain radiating to back, vomiting, and shortness of breath for one day. This case highlights ascariasis as a probable differentials diagnosis of acute pancreatitis in regions where worm infestation is common.

## Case presentation

According to the patient, he developed pain in epigastric and left upper quadrant region for one day. The pain was sudden in onset, sharp and severe in intensity, radiating towards the back, aggravated by taking meal and had no relieving factor. The pain was associated with vomiting since day one, sudden in onset, 4-5 episodes per day which was non-projectile, non-bilious, watery in consistency, and did not contain any blood. Vomiting was aggravated by taking meal. He also had complaints of non-exertional shortness of breath without associated chest pain. The patient had high grade fever of 101°F. The fever was continuous with no associated chills or rigors or special time of occurrence. There was not any history of associated sore throat, ear or nasal discharge, chest pain, cough, diarrhea, constipation, burning micturition, rashes, joint pain, weight loss, insect or mosquito bite. Review of the systems was thus non-significant. He had no past medical history of diabetes mellitus, ischemic heart disease, lipid profile abnormalities, tuberculosis or rheumatic disease. The patient had no blood transfusion history, no food or drug allergies. His family history was non-significant. His bowel habits were normal. His sleep was disturbed and appetite was decreased since the pain started. He gave no history of smoking, alcohol intake, or drug addiction. He was a rickshaw driver and usually ate at local restaurants. He belonged to lower socioeconomic class.

On presentation, the patient was anxious, had mild jaundice, but well oriented to time, place, person, with normal range vital signs except 92% SaO_2_ on pulse oximeter. Upon physical examination, abdomen had normal shape and symmetry, no scar marks, dilated veins, bruises or rashes. Upon palpation there was marked tenderness on superficial and deep palpation in the epigastric and left upper quadrant. No rigidity, shifting dullness, fluid thrill or visceromegaly was appreciated. Bowel sounds were audible in all four quadrants upon auscultation. Upon inspection, chest was of normal shape and symmetry. No deformity, scar marks, bruises or rashes were observed. Chest expansion was normal and symmetrical bilaterally. There was no tenderness upon palpation and vocal fremitus was normal. Percussion was tympanic with no hyper resonance or dullness except at lung bases. Upon auscultation he had normal vesicular breathing, except at lung bases bilaterally where breath sounds were mildly reduced, otherwise no wheeze or crepitations were heard. Cardio-vascular, neurological and musculoskeletal examinations were non-significant and revealed no abnormalities. No lymphadenopathy was appreciated. Probable differential diagnosis of gastritis, pancreatitis and cholecystitis was made. He was admitted for symptomatic treatment of pain, vomiting and dyspnea.

Lab investigations were significant for increased serum amylase and lipase as follows: serum amylase 1754 mg/dl; serum lipase 1272 U/L; complete blood count (CBC) showed leukocytosis, WBC 18 x 10^3^/µL (Table 1). Eosinophilia was not seen.

**Table 1:** Lab results on day 1 of admission. LDH: Lactate Dehydrogenase; SGOT: Serum Glutamic-Oxaloacetic Transaminase; SGPT: Serum Glutamic-Pyruvic Transaminase; Gamma GT: Gamma-Glutamyl Transferase; WBC: White Blood Cells.

Test	Result	Normal Range
Serum Amylase	1,754 mg/dl	<90 U/L
Serum Lipase	1,272 U/L	<50 U/L
LDH	1127 U/L	230-460 U/L
Total bilirubin	3.2 mg/dl	0.1-1.0 mg/dl
SGOT	439 U/L	<40 U/L
SGPT	311 U/L	<40 U/L
Alkaline phosphatase	383 U/L	<275 U/L
Gamma GT (GGT)	470 U/L	<46 U/L
WBC	18.7 x 10^3^/µL	4-10 x 10^3^/µL

Arterial blood gas test (ABG) result showed: pH 7.40 (7.35-7.45); PCO_2_ 36 (34-45 mmHg); PO_2_ 46 (70-100 mmHg); SaO_2_ 89% (90-100%); HCO_3_ 21.6 (22-28 mmol/L); Hb 12.6 (12-17 g/dL); Hct (c) 38 (37-50%); calcium ionized Ca^++^ 0.92 (1.12-1.32 mmol/L) (Table 2).

**Table 2:** Arterial blood gases. pH: Potential of hydrogen; PCO_2_: Arterial pressure of carbon dioxide; PO_2_: Arterial pressure of oxygen; SaO_2_: Arterial oxygen saturation; Na^+^: Sodium ion; K^+^: Potassium ion; Ca^++^: Ionized calcium; Hb: Hemoglobin; Hct: Hematocrit.

Arterial blood gases (ABGs)	Result	Normal range
Acid/Base/Oxygen status		
pH	7.40	7.35-7.45
PCO_2_	36 mmHg	35-45 mmHg
PO_2_	46 mmHg	70-100 mmHg
SaO_2_	89%	90-100%
Electrolytes		
Na^+^	139 mmol/L	135-145 mmol/L
K^+^	3.5 mmol/L	3.5-5.1 mmol/L
Ca^++^	0.92 mmol/L	1.12-1.32 mmol/L
HCO_3_	21.6 mmol/L	22-28 mmol/L
Hemoglobin		
Hb	12.6 g/dL	12.0-17.0 g/dL
Hct (c)	38%	37-50%

Serum electrolytes, urea, creatinine, fasting blood sugar, and lipid profile were normal. Hepatitis B and C serology tests were negative. HbA1c was 6.03%. Chest X-ray showed very mild bilateral pleural effusion. A probable diagnosis of acute pancreatitis was made based on the history, physical examination and initial lab results.

**Figure 1:**
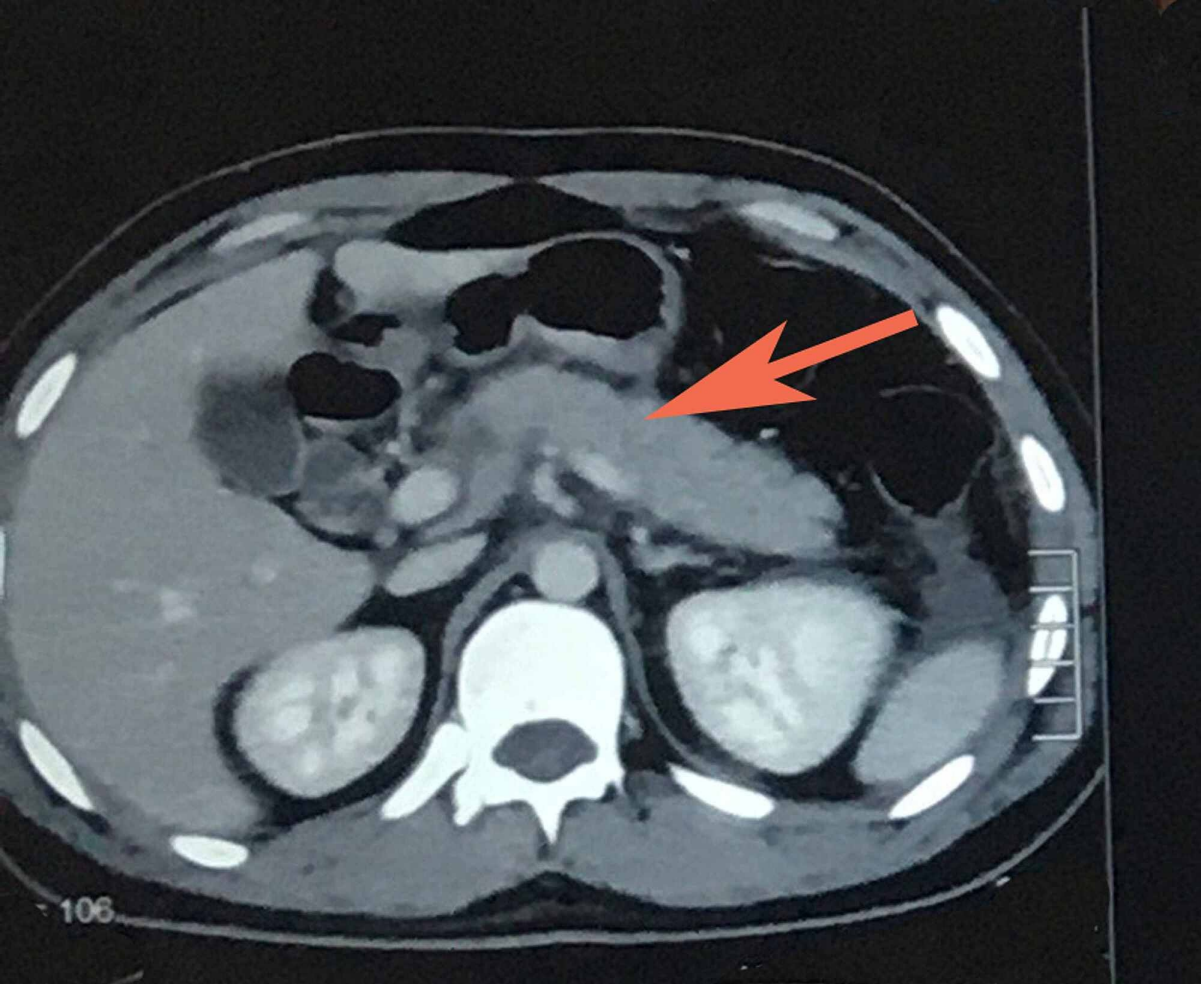
CT abdomen showing pancreatic inflammation highlighted by red arrow, bulky pancreas with peri-pancreatic fat stranding.

Ultrasound abdomen was done and showed normal study of liver, gallbladder, kidneys, spleen, and urinary bladder however could not comment on pancreas. Due to high degree of clinical suspicion, increased amylase and lipase, CT scan abdomen was done (Figure 1). Results showed bulky pancreas with significant peri-pancreatic fat stranding. No evidence of pancreatic necrosis or pseudocyst formation was seen. Mild ascites with mild bilateral pleural effusion and underlying atelectasis, more marked on the left side. Liver was mildly enlarged measuring 18 cm without any focal lesion. No evidence of intrahepatic or extrahepatic biliary dilation. Portal veins and hepatic veins were normally enhancing.

The case was further investigated for different etiologies of acute pancreatitis [3]. CT scan did not reveal any gall stone, hepatobiliary tumors, stenosis or congenital anomalies, evidence of intrahepatic or extrahepatic biliary dilation. His history was negative for alcohol intake, trauma, insect bite, steroid or diuretic intake, mumps, scorpion bite. He was not taking any other medication or drugs. He gave no history of diabetes mellitus, hypertriglyceridemia, hypercalcemia, hepatitis B or C, all of which were supported by normal blood tests. Routine exhaustive investigations for infectious etiologies were not performed neither they are advised due to lack of clear cause effect relationship between infections and pancreatitis and multiple infectious etiologies. Furthermore, patient’s history and physical examination did not support any infectious etiology such as mumps, Coxsackie B virus, Cytomegalovirus, Epstein-Barr virus, Herpes simplex virus, Varicella zoster virus.

Management

**Figure 2:**
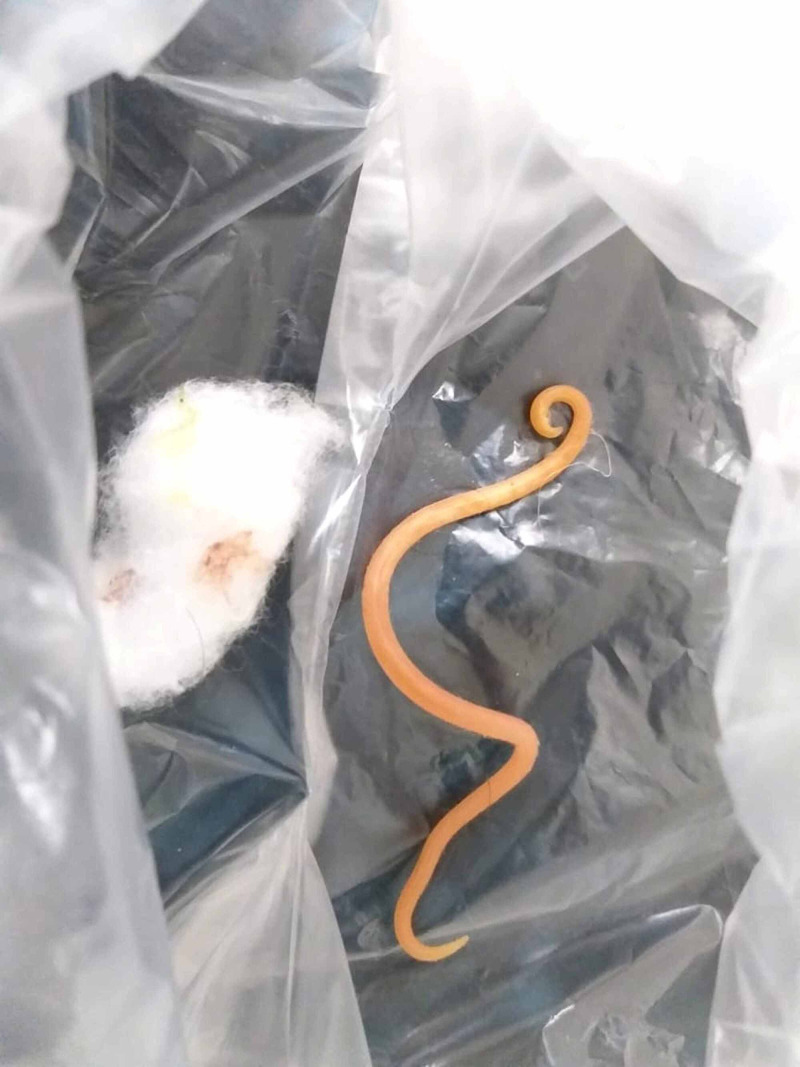
Round worm vomited by the patient.

Endoscopic-retrograde cholangiopancreatography (ERCP) and magnetic resonance cholangiopancreatography (MRCP) were not performed due to non-affordability and non-availability. Due to intolerance to oral intake he was passed nasogastric tube and urinary catheter. Temperature, pulse, respiration rate, and intake output chart were maintained. The patient was hydrated with intravenous fluids. Pain and nausea were treated with analgesics and anti-emetics. He was given high flow nasal oxygen for his dyspnea. He also received broad spectrum antibiotic due to fever and increased leukocyte count. However, the blood cultures came out negative and antibiotic was later stopped. On 4th day of admission, nasogastric tube was removed as his tolerance to oral intake had slightly improved. His serum amylase and lipase levels were showing very little improvement. On 6th day of admission, he vomited which contained, to everybody’s surprise, the round worm measuring 15 cm (Figure 2). Thereafter, he improved dramatically and his serum amylase and lipase levels returned to normal range with each passing day. His pain, vomiting and dyspnea improved significantly. At this point we ordered stool analysis for ova and parasite which was found negative in the first stool sample. However, the third stool sample was positive for ascariasis. Tablet Albendazole 400 mg once a day was recommended. Since anti-helminthic medicine works against adult worms not the larvae stage, the patient was discharged and planned for follow-up, after completion of treatment in two to three months. He would be re-evaluated by repeat history, physical examination, lab tests including stool analysis for ova and parasite.

## Discussion

Gallstone and alcohol abuse are the two major causes of acute pancreatitis [3]. History, lab works, and CT scan results of our patient all suggested pancreatitis. The revised Atlanta classification requires “that two or more of the following criteria be met for the diagnosis of acute pancreatitis: (a) abdominal pain suggestive of pancreatitis, (b) serum amylase or lipase level greater than three times the upper normal value, or (c) characteristic imaging findings. Contrast material-enhanced CT is most commonly used to fulfill the radiologic criterion, but magnetic resonance (MR) imaging is also appropriate” [4]. Our patient met the criteria mentioned for acute pancreatitis. It is pertinent to stress here that radiologists should be vigilant for radiographic signs of round worm in the gastrointestinal tract, such as echogenic tubular sign- strip sign on ultrasound, or relevant ERCP findings (smooth linear filling defect within the bile duct), MRI findings (T2 linear filling defect with associated pancreatic duct dilation) [5].

Ascariasis is one of the rare causes of pancreatitis and symptoms vary based on the region affected. As mentioned in the case presentation patient’s history, physical examination, lab works and CT scan had ruled out the possible etiologies. Ascariasis as the possible etiology was suspected when the patient vomited which contained the worm. He showed marked improvement thereafter. Although his first stool sample was negative, the third stool sample came out positive. Thus, it is strongly hypothesized that given the clinical and lab results improvement and prior reports of ascariasis-induced migration in the pancreatic duct, the causes of the patient’s symptoms were likely from worm infestation. However, an alternate etiology for pancreatitis could not completely be ruled out such as idiopathic pancreatitis. Round worm is on the list of etiologies in regions where helminthic infection is common. The suggested mechanism by which worms cause pancreatitis is through migration and obstruction of the pancreatic duct. In one of his research articles, Khuroo et al. writes, “they have a propensity to explore the orifices and in duodenum, the organism repeatedly enters into and out of the orifice of ampulla of Vater. The adult worm blocks the ampullary orifice and obstructs both the bile and the pancreatic duct. In addition, the writhing movements of the worm excites marked sphincter spasm and dysmotility” [1]. In his study, Klimovskij et al. writes, “presentations of forms are biliary colic (56%), acute cholangitis (25%), acute cholecystitis (13%) and acute pancreatitis (6%), and, rarely, hepatic abscess or haemobilia. Furthermore, there are reports of duodenal perforation caused by ascariasis” [2]. It is quite interesting that migratory and exploratory behaviour of the worm can cause a range of other gastrointestinal manifestations. In his article on ascariasis Khuroo et al. writes, “In the intestine it can cause intestinal ascariasis resulting in intestinal obstruction, bowel infarction and gangrene. In appendicular ascariasis it blocks appendix orifice causing appendicular colic, appendicitis, appendicular gangrene. Peritoneal ascariasis manifests as perforation, peritonitis, and septic shock. Ascariasis in the stomach and esophagus causes pyloric obstruction, nocturnal chocking as ascaris traversing into gullet at night, and unique retrosternal itching (ascarides in fundus and lower esophagus). Hepatobiliary and pancreatic ascariasis manifests as biliary colic, acute cholangitis, acalculous cholecystitis (choledochal or gall bladder ascariasis; ascarides in gall bladder may cause gall bladder gangrene), hepatic abscess (hepatic ascariasis). It also manifests as acute pancreatitis (duodenal ascariasis or pancreatic ascariasis, ascaride in pancreatic duct can cause necrotizing pancreatitis), hepatolithiasis (dead ascarides in hepatic ducts forming nidus of sludge/stones)” [1].

In a case report of ascariasis-induced pancreatitis ascariasis was reported as the etiologic agent in five (6%) of a series of 84 admitted patients [6]. As mentioned in article by Khuroo et al. “hepatobiliary and pancreatic ascariasis is a disease prevalent in endemic areas of world. Large series of patients have been published from several states of India including Kashmir, Kolkata, Assam, and several other endemic countries namely Saudi Arabia, Syria, Philippines and South Africa” [7]. However, to date no case has been reported from Baluchistan province of Pakistan. This is the first such case report from Baluchistan Pakistan. In fact very few cases have been reported from Pakistan. Thus, it is felt that it might be under diagnosed.

## Conclusions

Ascariasis is one of the rare causes of acute pancreatitis. It should be considered as one of the etiologies in geographical regions where ascariasis is common or sanitation and hygiene is poor. It is also recommended that the radiologist in the ascariasis endemic regions should be vigilant for radio-graphic signs of round worm presence in the gastrointestinal tract.
